# A Systematic Literature Review of E-Cigarette-Related Illness and Injury: Not Just for the Respirologist

**DOI:** 10.3390/ijerph17072248

**Published:** 2020-03-27

**Authors:** Anna Tzortzi, Melpo Kapetanstrataki, Vaso Evangelopoulou, Panagiotis Behrakis

**Affiliations:** 1 George D. Behrakis Research Lab, Hellenic Cancer Society, 17B Ipitou Street, 10557 Athens, Greece; vevan@researchlab.gr (V.E.); pbehrakis@acg.edu (P.B.); 2 Institute of Public Health, The American College of Greece, 17B Ipitou Street, 10557 Athens, Greece; 3 Pulmonary Department, Athens Medical Center, Distomou 5-7, Marousi, 15125 Athens, Greece

**Keywords:** electronic cigarette, vaping, e-vaping acute lung injury (EVALI), VAPI, e-cigarette explosion, nicotine intoxication

## Abstract

Following the recent electronic cigarette (e-cigarette) illness outbreak, the current review aimed to collect all related clinical cases for study and analysis and provide a critical synopsis of the proposed injury mechanism. Adhering to PRISMA (Preferred Reporting Items for Systematic Reviews and Meta-analysis) guidelines, e-cigarette-related clinical cases were identified via Google Scholar and PubMed databases. Additionally, references of published case reports and previous review papers were manually searched, revealing 159 publications presenting e-cigarette-related case reports and 19 reports by the Centers for Disease Control and Prevention. 238 individual cases were identified; 53% traumatic injuries due to e-cigarette explosion or self-combustion, 24% respiratory cases, and 12% poisonings. Additional cases pertained to oral, cardiovascular, immunologic, hematologic, allergic reactions, infant complications, and altered medication levels. Case reports were mainly published between 2016–2019 (78%). The oldest case, a lipoid pneumonia, was published in 2012. The current review showed that e-cigarette-related health effects extend beyond the acute lung injury syndrome, including traumatic, thermal injuries and acute intoxications. Physicians should be aware of the distinct clinical presentations and be trained to respond and treat effectively. Regulators and public health authorities should address the regulatory gap regarding electronic nicotine delivery systems (ENDS) and novel tobacco products.

## 1. Introduction

Introduced to the market in 2004 with the claim that the user inhales harmless vapor [1], the e-cigarette is marketed as a harm reduction product and has been proposed to be used as a smoking cessation tool, however there is a lack of clinical studies to support either of those effects [2]. 

Nearly two decades and four device generations later, there is growing scientific evidence that e-cigarette users are inhaling a mixture of irritative, toxic and carcinogenic compounds [3]. The device does not emit “side-stream smoke” as it is activated only by the user’s inspiratory effort; it does however produce secondhand aerosol (SHA) through the user’s exhalation [4]. SHA represents a documented source for passive exposure of bystanders as it contains micro-particulate matter (PM_10_ and PM_2.5_), volatile organic compounds and various other toxicants [5]. 

Following years of regulatory discussions, suggested policies and directives, e-cigarettes mainly targeting the youth continue to be underregulated especially outside the EU and UK; the easy online purchase and the customizable device and e-liquid mixture further complicate matters [6]. Regardless of their smoking status, adolescents tend to experiment with the nicotine containing «gadget» which predisposes them to addiction and smoking initiation [7]; e-cigarette users among high school students in the USA increased from 11.7% in 2017, to 20.8% in 2018 [8]. 

While the possible long-term health implications remain to be determined, studies showing immediate adverse health effects are mounting up. What has started as a small scale of laboratory-based studies on cells, tissues, animals, and humans [9], has evolved into a series of clinical case reports, including acute lung injury, poisoning, allergies, explosion accidents, and burn injuries [10]. 

The first case of e-cigarette-related lung injury was a case of lipoid pneumonia published in 2012 [11], furthermore from 2012 to 2015, 277 incidents of poisoning have been reported to National Poison Centers of 10 EU MS [12] and similarly increased poisoning incidents have been reported by US poison centers [13].

While in some countries, including the UK, the e-cigarette is used as a harm reduction product to aid smoking cessation, the severity of the 2019 acute lung injury outbreak in USA, mainly affecting adolescents and young adults was unanticipated. Having been attributed the term E-Vaping Acute Lung Injury (EVALI), earlier named Vaping Associated Lung Injury (VpALI) or Vaping Associated Pulmonary Injury (VAPI), and also an International Classification of Diseases (ICD) 10 coding [14], the syndrome continues to be under intense scientific scrutiny and diagnostic investigations, as the responsible causal factor and the underlying injury mechanism is not yet clear. On October 2019 the Centers for Disease Control and Prevention (CDC) issued a report which called for increased physician awareness, recommended the best approach of possible cases, and set the following criteria for EVALI diagnosis: “Vaping” or “dabbing” within 90 days prior to symptoms, pulmonary opacities on chest radiograph or ground-glass opacities on chest computerized tomography (CT) scan, negative infectious and immunologic panel, exclusion of alternative diagnoses [15,16]. CDC report issued in January 2020 showed that vitamin E acetate was identified as a possible causal factor for EVALI, however the contribution of other chemicals is yet to be determined [17]. 

To the authors knowledge at the time of publication there are 7 reviews on e-cigarette-related health effects and case reports, each specifically focusing on one aspect of the e-cigarette-related injury: The respiratory effects [18,19], poisonings [20], burn injuries [21,22], radiologic appearance of lung injuries [23], and histologic findings [24]. The majority of those reviews were published before the 2019 e-cigarette-related injury outbreak. The most integrated review including case reports referring to various organ-systems was published in 2016 [10] prior to the recent outbreak and the most recent in September 2019 [19] presenting mainly respiratory cases, toxicology of e-liquids, as well as previous mainly laboratory based, in vitro, in vivo, and animal studies. 

Considering the lack of an integrated up to date systematic literature review of clinical cases expanding across all medical disciplines, the aim of the current review was to collect all e-cigarette-related case reports for a comprehensive study and analysis, followed by a critical synopsis of the proposed injury theories, in an attempt to better understand the multifactorial process and possible mechanisms implicated in the etiology of the e-cigarette-related illness and injury.

## 2. Materials and Methods 

Adhered to the PRISMA guidelines [25] the current study aimed to systematically review published case reports of e-cigarette-related illness and injury. 

To identify published case reports we searched PubMed and Google Scholar databases without setting a time frame, in order not to miss the earliest of publications. The terms used were “e cig report”, “electronic cigarette report”, “e cig irritation”, “electronic cigarette irritation”, “e cig inflammation”, “e cig pneumonia”, “electronic cigarette pneumonia”, “e cig allergy”, “electronic cigarette allergy”, “nicotine intoxication”, “electronic cigarette respiratory effect”, “e cig cardiovascular”, “nicotine poisoning”, “e cig respiratory effect”, “nicotine salts”, “juul case report”, “pod case report”, “electronic cigarette health effect”, “electronic cigarette inflammation”, “electronic cigarette case”, “vape case report”, and “electronic cigarette review”. Cases identified from the references of case reports and previous review papers were manually searched in addition to articles found through specifically created alerts. Finally, all CDC reports regarding e-cigarette-related illness outbreak were searched.

### 2.1. Inclusion and Exclusion Criteria

Screening: The initial approach comprised of screening titles and abstracts, excluding papers presenting experimental and laboratory studies, animal, cellular and tissue studies and observational studies. 

For inclusion we considered publications in the English language presenting e-cigarette-related case reports including papers, systematic literature reviews, conference abstracts, letters to the editors/correspondence and CDC reports, provided they were published in peer-reviewed journals.

Eligibility: Following screening, 2 of the authors separately studied the full manuscript of the selected papers. Each case report was evaluated for its clinical presentation (quality, information abundance, e-liquid composition) and documentation of e-cigarette causal implication. Cases considered non-eligible by both authors were excluded. Cases deemed of moderate to low quality were further evaluated by two additional researchers and those deemed of poor quality were excluded. Cases with comorbidities relevant to the diagnosis regardless of the e-cigarette use were excluded. We included however cases where comorbidities and e-cigarette use may have had an equal or additive contribution to the reported diagnosis. 

### 2.2. Search Results

**Figure 1:**
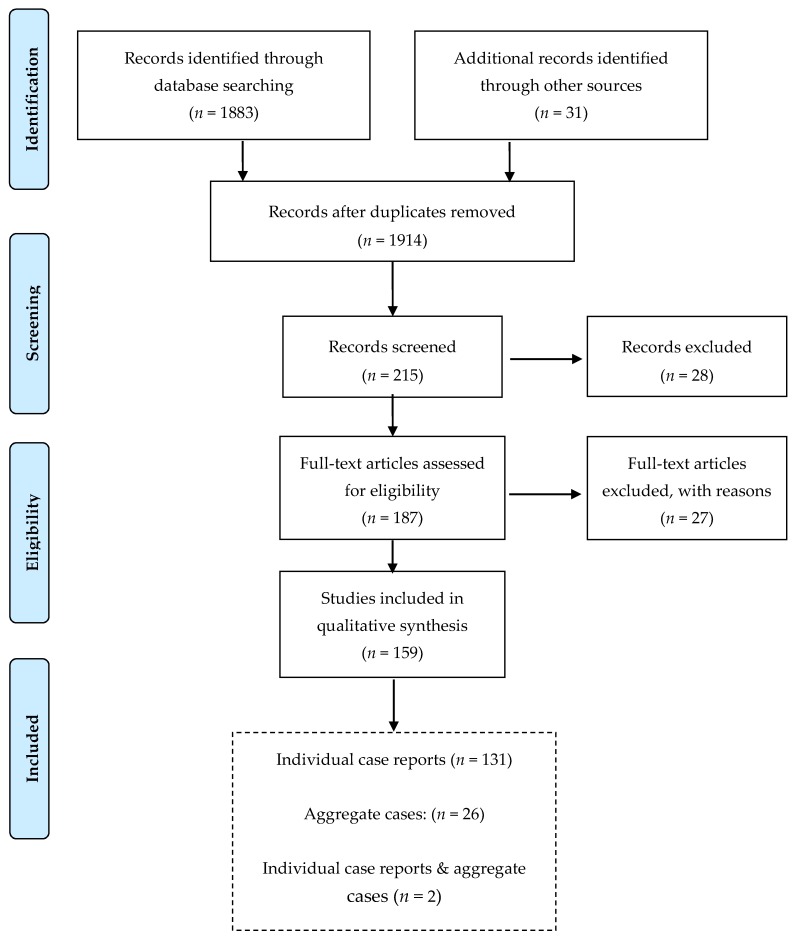
Flow chart of the process to identify and screen published case reports of e-cigarette-related illness and injury.

Up to 21 February 2020, our search resulted in 1914 papers regarding e-cigarettes in total, of which 215 presented case reports. Following the abovementioned inclusion criteria, our final dataset consisted of 159 papers (Figure 1). Additionally, 19 CDC reports were identified and will be presented separately.

### 2.3. Statistical Analysis

Descriptive analysis of the selected case reports was performed in Stata 13 (StataCorp. 2013. Stata Statistical Software: Release 13. College Station, Texas, TX 77845, USA: StataCorp LP). Results are presented as percentages or frequencies.

## 3. Results

### 3.1. Classification of Cases

**Figure 2:**
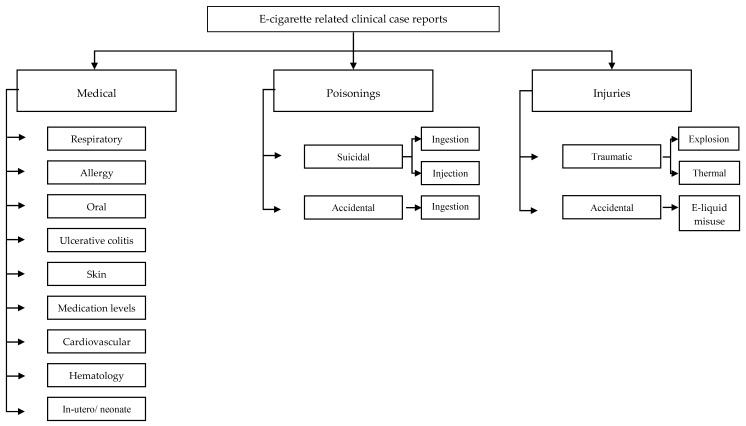
Classification by type of injury of e-cigarette-related case reports.

Selected case reports were classified in medical, poisonings and traumatic injuries. Medical cases were further categorized into respiratory, cardiovascular, allergic, autoimmune, and effect on medication metabolism among others; poisoning was further classified in accidental and suicidal and injuries in explosions and burns (Figure 2).

We identified 133 publications presenting 238 individual cases including 91 papers, 25 letters, 7 conference abstracts, 6 correspondences, 1 brief report, 1 conference paper, 1 short communication, and 1 publication presented as “Massachusetts General, Interesting Case Series”. Majority of publications (63%) pertained to cases in the US while the remaining 37% were from the UK, Australia, Canada, China, Denmark, France, Germany, Ireland, Italy, Japan, Korea, Malaysia, the Netherlands, Poland, Portugal, Scotland, South Korea, Switzerland, and Turkey (Table 1). Case reports were mainly published between 2016–2019 (78%), although there was a scattered number in the years 2013-2015 and 2020. The oldest case report identified was a lipoid pneumonia published in 2012. Two of the abovementioned publications in addition to the individual patients also presented aggregate cases. Twenty-six further publications presenting only aggregate cases on e-cigarette adverse health effects were identified (Figure 1).

### 3.2. Respiratory

41 publications were identified, presenting 58 respiratory cases. Main findings are presented in Table 2 and Table 3. Most common diagnosis was EVALI (*n* = 15) [26,27,28,29,30,31,32,33,34,35,36] or EVALI with an additional finding (*n* = 1) [37]. Second most common diagnosis included either organizing pneumonia/ Bronchiolitis obliterans with organizing pneumonia (BOOP)/ respiratory bronchiolitis (*n* = 12) [38,39,40,41,42,43,44,45,46] or lipoid pneumonia (*n* = 9) [11,47,48,49,50,51]. In 4 cases vaping precipitated a pneumothorax [52,53,54] and in 2 exacerbated pre-existing asthma [55]. Other diagnoses included eosinophilic pneumonia (*n* = 4) [56,57,58,59], combination of organizing and lipoid pneumonia (*n* = 3) [40], hypersensitivity pneumonitis (*n* = 3) [46,60,61], diffuse alveolar hemorrhage (DAH) (*n* = 1) [62], acute respiratory distress syndrome (ARDS) (*n* = 1) [63], a combination of ARDS, organizing pneumonia and diffuse alveolar damage (DAD) (*n* = 1) [64], epiglottitis (*n* = 1) [65], and a possible EVALI on asthma grounds (*n* = 1) [46]. 

Most patients were previously healthy (38/58). The majority were male (40/58) with median age 23 years old and interquartile range (IQR) 19–33 years old. The youngest person presented in the reports was 14 y.o. and the oldest person was 64 y.o. For the majority of cases it was not specified if they were dual users or if they used the e-cigarette for cessation (72%). While for 40% (23/58) of the cases it was not specified the substance used, 21 of cases used cannabis products solely, 6 used cannabis and nicotine in combination, 6 used cannabis and unknown liquid, while 2 used solely nicotine. Most common clinical symptom was dyspnea (48/58), cough (34/58), their combination (dyspnea and cough) (31/58) and fever (23/58). Sixty percent of patients (35/58) presented elevated white blood cell count (WBC), 7 patients had a normal WBC count while for 16 patients’ information was not provided. Information on infectious panels was available for 42 cases, all of which were negative. CT scan results were available for most cases (52/58), with most common finding being ground-glass opacities (GGO) (20/52) or GGO with consolidation (6/52). In total, GGO was mentioned in 37 cases. Ten cases required high flow nasal cannula (HFNC), 17 intubation/mechanical ventilation and 8 extracorporeal membrane oxygenation (ECMO) (Table 2). Bronchoscopy was performed in 43 cases; in 18 cases bronchoalveolar lavage (BAL) was positive for lipid-laden macrophages (LLMs), including Oil Red O Staining in 14 and 4 without Oil Red O Staining information. For 17 cases transbronchial biopsy was performed, 5 of whom were diagnosed with organizing pneumonia. For an additional 3 cases open lung biopsy was performed (Table 3). Majority was treated with corticosteroid administration (40/58) while 24 cases were also given antibiotics. Majority of patients recovered and were discharged home (*n* = 48), two patients were discharged but hospitalized again for asthma exacerbation, one left against medical advice however he was re-hospitalized, and 6 presented persisting abnormalities in lung function tests, long-term rehabilitation, psychiatric care. Additionally there was one fatality.

Additionally, 8 publications presenting respiratory aggregate cases were identified. A total of 104 cases with EVALI symptoms cared for in the authors’ institutions were presented in 6 papers [24,66,67,68,69,70], while in one publication [42] the focus was on Illinois and Wisconsin patients (53 cases) and in another publication [71] 60 EVALI patients admitted to 24 hospitals of Utah, Idaho, Wyoming and Nevada were presented.

### 3.3. CDC Reports on Respiratory Cases

Since the EVALI outbreak, CDC has published 19 reports in an effort to provide guidance to health care professionals and assist them identify patients with EVALI symptoms [15,72,73,74], to describe the patients’ characteristics [17,75,76,77,78,79,80,81,82,83] and to identify possible risk-factors associated with EVALI [6,84,85,86,87]. According to the most recent CDC report published on the 24th January 2020 [87], 2668 EVALI patients have been hospitalized in the USA and reported to the CDC until the 14th January 2020.

### 3.4. Traumatic Injury

42 publications presenting 126 cases of injury were identified. The majority of injuries were caused due to explosion (82/126), including 48 assembled devices [21,88,89,90,91,92,93,94,95,96,97,98,99,100,101,102,103,104,105,106,107,108,109,110,111,112,113,114], 20 self-exploded batteries [95,97,102,111,112,115,116,117] and 14 without specifying [118]. Second most common injury was thermal burns (24/126) caused by 14 self-combusted batteries [21,111,115,119,120,121,122], 6 self-combusted assembled devices [115,120,123,124], 2 exploding devices [99,102], 1 in-pocket e-cigarette self-activation [125] and 1 case for which the ignition was induced by a motorcycle crash [111]. A combination of explosion and thermal burn caused by the explosion of the assembled device (7/126) [21,112,120,126,127] was the 3rd most common cause of injury. In 1 case their injury was caused by a flash burn [93] and in 12 it was not specified [128]. The type or generation of the device was not provided in the vast majority of papers.

The vast majority of cases were in the USA (99/126), male (120/126) with a median age of 28 years old. Most common affected body areas were the thighs (85/126) followed by hands (49/126). Twenty-three cases sustained facial injuries, with three including eye injuries. In total 55 cases sustained injuries in multiple body areas. Information on total burn surface area (TBSA) was given for the majority of cases (101/126), with median TBSA 4% (IQR: 2–6%). Skin grafting was performed in 40 patients in total, including 25 who required both skin grafting and excision, 1 finger amputation, and 2 foreign body removals, while 18 patients required minor surgical procedures. The majority of cases (105/126) were discharged with no further complications while for 6 cases there was no discharge information provided. For the rest 15 cases complications included amputation (1/15), back pain (2/15), post-traumatic stress disorder (PTSD) (1/15), pain and scarring (1/15), scarring (2/15), photophobia (1/15), eye (2/15), teeth (1/15), and neurologic (3/15) complications, as well as discharge to burn center (1/15).

Additionally, 10 publications presenting aggregate cases with traumatic injury were identified: 5 publications [129,130,131,132,133] presented a total of 86 cases from the authors’ institutions; 3 publications [90,134,135] presented a total of 311 cases from the USA (however the total number of cases could be overestimated as those publications could have some mutual cases presented). Finally, 2 additional publications [136,137] presented USA burn centers cases estimated as 2035 in 2015–2017 and 1007 in 2016. 

### 3.5. Poisoning

Twenty-five papers presenting 28 cases of nicotine poisonings were identified. Interestingly, publications were not limited to the USA but were scattered around the world (6 from the USA, 3 from Korea, 2 from each of the UK, Italy and Germany and 1 from each of Poland, Denmark, Canada, China, France, Japan, the Netherlands, South Korea, Switzerland, and Turkey). 

Poisonings were caused by accidental (9/28) [138,139,140,141,142,143,144,145,146] or intentional ingestion (14/28) [147,148,149,150,151,152,153,154,155,156,157,158] of e-liquid, intravenous injection of e-liquid (4/28) [151,159,160,161], or both ingestion and injection (1/28) [162]. 

Accidental ingestion was observed only to young children with a median age of 2 years old (IQR: 0.85–4) and mainly to females (6/8). Information on sex was not available for 1 case. Nicotine concentration was available for 3 cases, with nicotine ingested being 8.2 mg, 50 mg, and 60 mg. Two children required admission to the intensive care unit (ICU) and intubation, while most children did not require an invasive treatment (5/9). The most severe outcome was death (2/9), followed by hearing complications (1/9), while the majority of cases were discharged home with no complications (6/9).

Suicidal attempts (i.e., ingestion and injection of e-liquid) were mainly by adults with a median age of 27 years old (IQR: 22–36). Majority of cases were male (13/19). Total nicotine intake was available for 8 cases, ranging from 2100 mg to 128.8 mg. ICU admission was required in 8 cases, including 3 intubations. Information on treatment was available for 9 cases with most common being the administration of activated charcoal (5/9). Eight cases were discharged without complications, 4 cases were reportedly improved without further information, 1 case remained semi-comatose without awareness (Cerebral Performance Category 4), and 6 were fatal (3 deaths at hospital and 3 at the scene).

Additionally, 10 papers presenting aggregate data on poisonings were identified. Between 2010–2013 the USA Poison Centers received 1700 calls regarding exposures to electronic cigarettes [13], between 2010–2014 the calls were 2405 [163] whereas between 2010–2018 the calls were 17,358 [164]. In Texas, Poison Centers received 225 calls between 2009–2014 [165], while in Utah 52 cases were reported to have been poisoned by a synthetic cannabinoid in 2017–2018 [166]. Between 2012–2015, 277 calls were made to 10 European Countries’ Poison Centers [12], while in the UK, between 2008-2016, 278 calls were made to the National Poisons Information Service (NPIS) regarding children under 16 years old [167]. Between 2012–2018, 148 cases of acute exposure to e-cigarettes were reported to Czech Toxicological Information Centre [69]. Finally, 2 publications presented an estimate on the poisonings of under 5 year-olds between 2013–2017 (4745 cases) [168] and in 2018 (885 cases) [169].

### 3.6. Allergy

Four publications presenting 5 cases of allergic contact dermatitis to nickel were identified [170,171,172,173]. Majority of patients were female (3/5), in their late 30s or early 50s. For all cases the dermatitis was treated with the avoidance of the e-cig.

### 3.7. Effect on Medication Metabolism and Plasma Levels

We identified 2 publications presenting 2 cases for which the use of the electronic cigarette increased their clozapine levels [174,175] and 1 publication presenting a patient with epilepsy for whom it increased their seizure frequency [176]. Two of the patients were 16 years old and 23 years old females and there was one 52-year-old male.

### 3.8. Ulcerative Colitis

We identified 2 publications presenting 2 cases for whom the e-cig was associated with their ulcerative colitis. The first was a 2013 publication [177] from the USA presenting the case of a 35-year-old male for whom their ulcerative colitis improved after the initiation of an electronic cigarette. The second was a 2014 publication [178] from France, presenting a 49-year-old female smoker for whom their ulcerative colitis reappeared after switching from combustible to electronic cigarette.

### 3.9. Misuse of E-liquid

Accidental misuse of e-liquid was presented in 2 publications [179,180]. Two case reports (a 50-year-old and a 32-year-old female) were presented, who have mistaken the e-liquid bottle for eye-drops. Both had immediately realized their mistake and washed out the liquid before attending the emergency department (ED). Treatment was described for one of the cases for whom their misuse resulted in corneal burn, including eye irrigation, analgesics, anti-inflammatory and antibiotic eye-drops. 

### 3.10. Injury Caused by Falling with E-cigarette in Mouth

A case report of a male in the USA who fell while he had his e-cigarette in his mouth [181] was presented in a letter published in 2018. The age of the patient was not published. The reason of the fall was speculated to be due to loss of consciousness resulting in ICU admission, tracheostomy, intraoperative examination, esophagogastroduodenoscopy and feeding tube placement, which was needed even at 6-month follow-up. 

### 3.11. Additional Diagnoses and Health Effects Attributed to Electronic Cigarette Use

Thirteen publications were identified; 4 oral cases including a lingua villosa nigra [182], a lichenoid eruption [183], a necrotic ulcer [184], and an acute uvulitis [185], 2 cases with skin-grafts compromise [186,187], 2 cases of coronary events [188,189] in 16 and 24 year-old males, 1 case of neonatal necrotizing enterocolitis due to in-utero exposure [190], and 1 case of polycythemia [191]. Finally, in one case the e-cigarette use was attributed an anti-inflammatory [192] and in 2 cases an antibacterial [193,194] effect.

Detailed information for each case report included in the current review is presented in the Supplementary Materials Table S1: “E-cigarette related case reports by type of injury”.

## 4. Discussion

The shift towards novel noncombustible products has significantly altered the diagnostic algorithm by introducing to clinical practice new risk factors for adverse health effects.

The current review is the first to show the potential of e-cigarette use to lead not only to acute and severe lung injury syndromes, but also to acute poisonings, traumatic injuries and to interfere with medication bioavailability, in addition to providing a new vehicle for the inhalational abuse of several psychoactive medications and recreational drugs that may easily be added in the e-liquid.

Three major categories of cases were reported: medical, poisonings and injuries. Majority of respiratory cases were associated with Tetrahydrocannabinol (THC) use, whereas, cases of acute poisonings were mainly associated with nicotine use.

### 4.1. Respiratory Injuries

Majority of reported cases referred to previously healthy adolescents and young adults, a finding of great significance as studies have shown that adolescents addicted to nicotine are predisposed to addiction to other substance/substances as well [195]. Users experienced a range of acute and severe lung injury syndromes, that led to hospitalizations, need for mechanical ventilation, use of ECMO and ultimately loss of life. 

In the majority of cases, the injury involved distal airway and parenchymal areas and the histologic findings of acute lung injury patterns, mainly DAD and organizing pneumonia, were consistent with an underlying inflammatory pathophysiology [39]. The inflammatory pathway response, possibly triggered by the inhalational exposure to e-cigarette aerosols, was expressed as various types of pneumonitis (lipoid, organizing, eosinophilic, hypersensitivity, interstitial) often complicated by ARDS [196].

No single causal factor has yet been identified for EVALI, however it is worth noting that most cases in the current review and over 80% of those reported to the CDC [87], have used cannabinoids. Vaping cannabinoid oils has been associated with lipoid pneumonia [29] and LLMs were detected in almost half (19/35) of the bronchoscopy specimens included in the current review.

Although there was not a consistent method of LLM staining, measurement and reporting, and their role in diagnosis of EVALI has been questioned by researchers [197], majority of lipoid pneumonia cases in this review complied with the diagnostic criteria [198], suggesting a possibly new risk factor for exogenous lipoid pneumonia [199].

The 6th report issued by CDC [84] was dedicated to discussing the lipoid pneumonia cases in association with vaping; diagnosis criteria used, included: Lipid-containing e-liquid use (such as marijuana oils), consistent imaging (CT scan/radiography), exclusion of differential diagnoses and presence of LLMs in BAL cytology preferably using oil Red-O or Sudan stain. 

Travis S. et al. [22], suggest that even non-oil e-liquid ingredients may potentially trigger the endogenous phospho-lipidosis mechanism and lead to an amiodarone like lung toxicity, therefore representing a risk factor for endogenous lipoid pneumonia as well. Furthermore, vaping has been causally associated with acute eosinophilic pneumonia similarly to smoking [200,201].

The current review also highlighted the adverse effect of vaping on asthma, which similarly to that of smoking, is translated in asthma exacerbation with potentially more frequent, severe, or difficult to control asthma attacks [54,202].

Pneumothorax cases, although pertaining to predisposed individuals (blebs and bullae on CT scan, compatible body type), indicate that vaping should be also considered as a risk factor for pneumothorax and emphysema. Cannabis vaping in particular, could have induce barotrauma, spontaneous pneumothorax and bullous emphysema similarly to cannabis smoking; the deep inhalation practiced by marihuana users has been proposed to possibly lead to more negative alveolar pressure and alveolar-capillary membrane injury [203]. Furthermore, the anticoagulant activity known to be exerted by cannabis could explain the occurrence of hemoptysis and diagnosis of DAH [204].

The etiology of organizing pneumonia includes infectious agents, medications, chemicals, and radiation, suggesting that exposure to an inhalational trigger originating from the e-liquid constituents and, or their degradation products, possibly diacetyl, could be the cause [205]. Diacetyl has been previously suggested as the cause of BOOP in diacetyl plant workers [206] and workers in microwave popcorn industry [207], while rats exposed in diacetyl inhalation developed airway epithelium changes, consistent with BOOP [208]; diagnosis requires evidence of exposure, exclusion of infection or other illness, compatible lung function tests, chest CT scan and ultimately lung biopsy, criteria that have been met by the case reports included in the current review.

Carl A. Vas et al. [209], in a study funded by British American Tobacco (BAT), showed that the use of acetoine in e-liquids leads to a continuous diacetyl formation even during storage time, a process dependent on the influence of light, nicotine concentration, increased e-liquid pH and levels of propylene glycol (PG)/vegetable glycerin (VG); therefore the authors suggest that acetoine addition to e-liquids should be avoided. Vitamin E acetate has been suggested as another possible toxicant especially for EVALI [86]. Vitamin E acetate was present in the majority of the bronchoalveolar lavage (BAL) samples collected from EVALI patients, who used THC, while it was not identified in the healthy controls; e-cig liquids containing THC, usually also contain various concentrations of vitamin E acetate which is used to dilute, “cut” THC [210].

Furthermore, studies on humans have shown that vaping altered nasal mucosa genes towards immune suppression [211], levels and expression of >200 bronchial epithelium proteins associated with membrane functionality, possibly through PG/VG [212], in addition to pulmonary lipid homeostasis and immunity alterations, through ingredients other than nicotine [213].

As of January 2020, 2668 hospitalized EVALI cases had been reported, with 82% of those having reported use of THC containing e-liquid [87]; vitamin E acetate was strongly associated with EVALI, however potential toxicant role of other ingredients has not yet been ruled out [83]. The causal role of each of the e-liquid ingredients, their thermolysis byproducts, potential interactions and additive effect, as well as the role of the patients’ immunological response to the ultimate injury expression and outcome, is the subject of ongoing scientific research [67]. Currently CDC recommends avoidance of THC-containing e-liquids and especially those originating from unauthorized/illicit sources [87]. 

### 4.2. Accidents

The majority of reviewed cases presented accidents leading to traumatic, chemical, and thermal injuries, mainly caused by technical/safety flaws, as the device and or the battery could self-explode. Self-explosion of the assembled device, or the “in pocket kept” battery, may explain why the majority of cases were men with thigh and or hand injuries; the warm and humid pocket conditions and the presence of metallic objects usually keys have been proposed as possible causes. 

Patterson [90] proposed a dual classification of burn injuries from e-cigarette explosions: direct/indirect and an additional arithmetic classification in types 1–5b: types 1, 2, and 3 were defined by the body area affected (hand, face, waist, and groin), type 4 included injuries from house fire, while type 5 included inhalation injuries from device on fire further subclassified in—5a (upper airway injuries from direct flash or explosion of the e-cigarette), and 5b (chemical, subglottic smoke inhalation injury). Types 1, 2, 3, and 5a were the direct injuries, whereas type 4 and 5b were the indirect ones from house or vehicle fire. Patterson then proceeded to indicate preventive measures to fit each specific type. 

### 4.3. Poisonings

In the pre-e-cigarette era, nicotine intoxication was rarely reported in humans, with the exception of tobacco manufacturing workers [214]. E-cigarette exposes user to novel risk factors for poisonings involving both adults and accidentally children and has brought to light the underestimated, often times ignored, nicotine, PG, and cannabis toxicity. In the cardiovascular category of this review the cases of two young adults were presented who experienced acute myocardial ischemia; as the presence of other risk factors was excluded, it was suggested that the cause was the effect of cannabis and nicotine in the e-liquid used respectively.

Case reports of nicotine poisonings gave a new insight in the metabolism, bioavailability and the dose/effect relationship of the substance [19,151], adding to the existing controversies regarding the level of fatal nicotine concentrations. The fatal dose of 60 mg indicated by Lazutka et al. in 1969 [215] was based on studies in mice, while more recent studies suggest an oral lethal dose of 0.5–1 gr [216].

Injection of nicotine and PG as contained in the e-liquid mixture, leads to a new type of acute intoxication, physicians should be aware of. PG intoxication leads to lactic acidosis with elevated anion gap, while acute nicotine intoxication presents in two progressively aggravating clinical phases; until the second more severe phase of Central Nervous System depression and respiratory failure supervenes, there is a 3-hour window opportunity for the physician to intervene [161].

E-liquid storage together with medications, or in empty bottles previously containing other products was the main reason for misuse, as it has been mistaken for eye drops or medical syrup. Opthalmologists and emergency personnel should be aware of the variable e-liquid pH which can be acidic [180] or alkaline [179] and treat the eye as indicated to restore a normal eye pH. 

Pediatric poisonings in particular, have increased alarmingly within the period 2010–2018 following the similarly increasing trend of e-cig use. Pediatric poisonings were accidental and majority of cases were treated without complications.

In contrast, adult poisonings primarily represented intentional suicidal attempts by ingestion and or injection; 1/3 of those suicidal attempts were successful.

Unmet device and package safety requirements such as child proof e-liquid bottle cap, the sweet, fruity e-liquid flavors, the various sizes of e-liquid vials and high nicotine concentrations, lie behind the accidental ingestion. Since 2016, in the EU and UK regulations regarding packaging with enhanced child-proof features protect the childhood population from undesired poisonings. Under the EU TPD, e-liquid vials should be limited to a maximum of 20mg/mL nicotine concentration, refill containers to a maximum of 10 mL size and tanks to a 2 mL size, thus containing 200 mg and 40 mg total nicotine quantity respectively [217]. However, outside the EU and through unauthorized sources over the web, refill mixtures may contain nicotine concentrations ranging from 18 mg/mL to 59 mg/mL, with the highest concentrations being contained by the newer device generations, especially those using protonated nicotine salts; moreover manipulation of the device’s wattage and voltage by the user may enhance the nicotine concentration in the delivered aerosol [8]. 

### 4.4. Substance Abuse

E-cigarette is used as a vehicle for inhalation of nicotine, cannabis, fentanyl and other psychoactive substances, either pure or combined in various mixtures; considering the addictive nature of those substances it becomes evident the potential of e-cig to function as a gateway to nicotine and various other addictive substances/drugs [17,218].

### 4.5. Seizures and Effect on Medication Metabolism

Rong et al., 2014 [219] concluded that smoking and epilepsy relationship is unclear, while Iha et al. [220] in their study in mice, suggested that nicotine activates certain brain regions such as the epileptogenic amygdala; indeed, nicotine injection to amygdala led to convulsive seizures, therefore supporting their hypothesis. Seizures are a known clinical feature of the acute nicotine intoxication. Cases reporting seizures in the current review, suggest that vaping induces seizures either through nicotine, either through PG and glycerol (G) induced circadian rhythm alterations [176]; the potential effect on medication levels should be also considered and therefore patients with epilepsy be informed accordingly.

Increased clozapine levels reported in the reviewed cases, practically highlight the effect of nicotine on medication plasma levels and raise wider concerns regarding potential interactions with other medications as well. Patients’ smoking and or vaping behavior should be extensively asked and specifically sought for by clinicians during history interview and more importantly, taken into account whenever changes of dosage or medication regimen are attempted. Furthermore, patients should be aware that even when switching between different tobacco products (i.e., combustible/e-cigarette and vice versa), or are planning smoking cessation, serum levels of their medication might be altered [221].

### 4.6. Regulatory Gap 

The current review presents the emergence of a novel public health risk that although associated with the use of tobacco products, goes beyond pulmonology and expands across several medical specialties, previously unlikely to be implicated. In addition, it reveals the existent regulatory gap [222] regarding the ENDS and highlights the need for more efficient, universal, protective and preventive measures. Majority of the ENDS regulations to date are limited to simple recommendations. Multilevel, universal regulations should be placed regarding the design, development and safety of the device and its components, the ingredients/flavors/additives, including their package safety and quantity limit, especially for toxic compounds such as nicotine.

### 4.7. Limitations

A limited number of e-cigarette-related cases was excluded during the screening process because of insufficient information provided by the authors of the respective publications. The claimed health-effect was not clearly related to the e-cigarette use. 

Another limitation derives from the fact that the current review focused on the e-cigarette-related clinical cases and published case reports, therefore experimental, observational studies and clinical trials were not included. Finally, worldwide public health organizations stands regarding e-cigarette regulation and regulation gaps as well as the harm reduction product and cessation tool approach regarding its use were not examined.

## 5. Conclusions

The current review is the first to show the full range of the e-cigarette-related injury which extends beyond the plausible respiratory disorder; in addition to the acute lung injury syndromes, it is also associated with accidents leading to traumatic and thermal injuries and severe, potentially fatal, acute intoxications. Physicians should be aware of the distinct clinical presentations and trained to respond and treat effectively. To protect and promote public health, regulators and public health authorities such as the European Commission, CDC, FDA, and WHO should address the regulatory gap regarding ENDS and novel tobacco products, aiming to protectively cover the global population.

## Figures and Tables

**Table 1:** Type of injury by geographical location.

Type of Injury	USA	UK	EU-Other	Other
Respiratory	36 (88%)	1 (2%)	-	4 (10%)
Traumatic injury	32 (76%)	5 (12%)	3 (7%)	2 (5%)
Poisoning	6 (24%)	2 (8%)	9 (36%)	8 (32%)
Allergy	1 (25%)	2 (50%)	1 (25%)	-
Effect on medication metabolism and plasma levels	2 (67%)	-	1 (33%)	-
Ulcerative colitis	1 (50%)	-	1 (50%)	-
Misuse of e-liquid	-	-	2 (100%)	-
Injury caused by falling with e-cigarette in mouth	1 (100%)	-	-	-
Additional diagnoses and health effects attributed to electronic cigarette use	5 (38%)	4 (31%)	2 (15%)	2 (15%)

**Table 2:** Respiratory cases: demographic, clinical, laboratory findings, and outcome.

Variable	*n* = 58
**Sex (%)**	
Male	69%
Female	31%
**Age in years median (IQR)**	23 (19–33)
**Dual user (e-cig+ combustible cigarette)**	
Yes	10%
No	16%
Unspecified	74%
**E-cig for cessation**	
Yes	13%
No	71%
Unspecified	16%
**Medical history (%)**	
No medical history	66%
Asthma/ Allergy	21%
Other *	14%
**Substances used (%)**	
CBD/THC	36%
CBD/THC and Nicotine	10%
CBD/THC and unknown liquid	10%
Nicotine	3%
Unknown/unspecified liquid	40%
**Symptoms (%)**	
Dyspnea	83%
Cough	59%
Dyspnea and Cough	53%
Chest pain	22%
Hemoptysis	9%
Fever	40%
Respiratory arrest	5%
Gastrointestinal symptoms	26%
**CT (%)—available for 52 cases**	
GGO	38%
GGO + consolidation	12%
Opacities	10%
Multiple nodules	6%
GGO + multiple nodules	6%
Other	28%
**Interventions**	
High flow nasal cannula therapy	18%
Intubation/ mechanical ventilation	31%
ECMO	15%
Bronchoscopy	78%
**Diagnosis**	
EVALI	26%
Organizing pneumonia/BOOP/Respiratory bronchiolitis	21%
Lipoid pneumonia	16%
Eosinophilic pneumonia	7%
Pneumothorax	7%
Hypersensitivity pneumonitis	5%
Organizing pneumonia and lipoid pneumonia	5%
Asthma exacerbation	3%
ARDS	2%
ARDS-DAD-Organizing pneumonia	2%
DAH	2%
EVALI and secondary pneumothorax	2%
Epiglottitis	2%
Possible EVALI on asthma grounds	2%
**Corticosteroid administration**	73%
Outcome	
Recovered	83%
Discharged but hospitalized again	5%
Persisting complications	10%
Deceased	2%

Abbreviations: CBD: Cannabidiol, THC: Tetrahydrocannabinol, GGO: Ground-glass opacities, ECMO: extra-corporeal membrane oxygenation, BOOP: Bronchiolitis obliterans with organizing pneumonia, EVALI: E-Vaping Acute Lung Injury, ARDS: acute respiratory distress syndrome, DAD: diffuse alveolar damage, DAH: diffuse alveolar hemorrhage. * Other medical history includes: Inflammatory bowel disease, Congenital dysmorphism with thrombocytopenia, anemia, Hashmimoto’s thyroid, diabetes, cancer, seizure disorder, and hypertension.

**Table 3:** Publications of e-cigarette case reports with cytologic and histologic findings.

Paper	Country	Bronchoalveolar Lavage (BAL)	Transbronchial Biopsy	Open Lung Biopsy
He et al., 2017 [38]	USA	Possible DAH	Organizing pneumonia	
Modi et al., 2015 [47]	USA	LLM (Oil Red O positive)		
Mantilla et al., 2016 [45]	USA	No cytology information	BOOP	
Mukhopadhyay et al., 2019 [40]	USA	Macrophage predominant	DAD (acute and organizing)	
		None obtained	Organizing pneumonia	
		LLM (Oil Red O positive)	Organizing ALI	
		LLM (Oil Red O positive)	Organizing pneumonia	
		LLM (Oil Red O positive)	Organizing ALI	
		Macrophage predominant	Organizing pneumonia	
		Macrophage predominant	Organizing pneumonia	
Arter et al., 2019 [56]	USA	26% eosinophils		
Agustin et al., 2018 [62]	USA	Recurrent DAH		
Sommerfeld et al., 2018 [60]	USA	LLM (Oil Red O positive)		
Khan et al., 2018 [41]	USA	Organizing pneumonia		
Flower et al., 2017 [43]	Australia	Non-diagnostic	Non-diagnostic	RUL: black pigmentation and bullae
Thota D and Latham E, 2014 [57]	USA	Negative cultures		
McCauley et al., 2012 [11]	USA	LLM (Oil Red O positive)		
Itoh et al., 2018 [33]	Japan	LLM (Oil Red O positive)	Acute alveolitis intra-alveolar fibrosis	
Dicpinigaitis et al., 2019 [51]	USA	LLM (Oil Red O positive)		
Landman et al., 2019 [39]	Canada	Negative cultures	Non-specific acute inflammation and reactive changes	
Viswam et al., 2018 [50]	UK	Pink cloudy fluid Negative microbiology and cytology		LLM and cholesterol clefts
Maddock et al., 2019 [49]	USA	49% neutrophils, >50% LLM (Oil Red O positive)		
		~50% LLM (Oil Red O positive)		
		~30% LLM (Oil Red O positive)		
		~75% LLM (Oil Red O positive)		
Layden et. al, 2019 [42]	USA	78% neutrophils, some LLM (Oil Red O positive)		
Sharma et al., 2019 [37]	USA	Lymphocytes predominance and rare eosinophils		
Pokhrel et al., 2019 [31]	USA	LLM predominance		
		Neutrophil predominance		
		LLM predominance		
Abeles et al., 2019 [28]	USA	51% PMN, negative cultures, rare LLMs		
Casanova et al. 2019 [30]	USA	55% LLMs		
Ocampo-Gonzalez and Park, 2019 [32]	USA	80% LLM (Oil Red O positive)		
Attis et al., 2018 [61]	USA	Macrophage predominance		
Aftab et al., 2019 [35]	USA	91% neutrophils		
Buus et al., 2019 [29]	USA	Macrophages/ neutrophils/ lymphocytes		
Lu et al., 2020 [44]	USA		Ιntra-alveolar fibrin and neutrophils	
Youmans et al., 2020 [64]	USA	50% monocytes, 40% lymphocytes, 10% neutrophils	Non-diagnostic	Acute and organizing DAD with foamy macrophages
Antwi-Amoabeng et al., 2020 [59]	USA	75% eosinophils		
Ansari-Gilani et al., 2020 [46]	USA	Unremarkable	Poorly define granulomas	
		Unremarkable		
Abbara et al., 2019 [48]	USA		Lipoid pneumonia	

Abbreviations: DAH: diffuse alveolar hemorrhage, LLM: lipid-laden macrophages, BOOP: Bronchiolitis obliterans with organizing pneumonia, DAD: diffuse alveolar damage, ALI: acute lung injury, RUL: Right-upper lobe, PMN: polymorphonuclear.

## Supplementary Materials

The following are available online at https://www.mdpi.com/1660-4601/17/7/2248/s1, Table S1: e-cigarette-related case reports by type of injury.xlsx.
